# Retraction Note: Analysis of the Thomson and Troian velocity slip for the flow of ternary nanofluid past a stretching sheet

**DOI:** 10.1038/s41598-024-83032-z

**Published:** 2024-12-17

**Authors:** Shuguang Li, V. Puneeth, A. M. Saeed, A. Singhal, Fuad A. M. Al-Yarimi, M. Ijaz Khan, Sayed M. Eldin

**Affiliations:** 1 School of Computer Science and Technology, Shandong Technology and Business University, Yantai, 264005 China; 2 Department of Computational Sciences, CHRIST University, Bangalore, India; 3 Department of Mathematics, College of Science, Qassim University, Buraydah, Saudi Arabia; 4 Department of Computer Science, King Khalid University, Muhayel Aseer, Kingdom of Saudi Arabia; 5 Department of Mechanical Engineering, Lebanese American University, Beirut, Lebanon; 6 Department of Mathematics and Statistics, Riphah International University, I-14, Islamabad, 44000 Pakistan; 7 Center of Research, Faculty of Engineering, Future University in Egypt, New Cairo, 11835 Egypt

Retraction of: *Scientific Reports* 10.1038/s41598-023-29485-0, published online 09 February 2023

The Editors have retracted this Article.

After publication, concerns were raised about the validity of the research presented in this Article. Specifically,

- two variables, *u*_2_* and *b*, in the unnumbered equation before the *Solution methodology* section are not defined;
- the references in Table 2 do not contain the data reported in the table. The comparison presented is therefore not valid;
- the results presented in Figures 2 through 9 do not exhibit any meaningful physical dependence on the parameters chosen.

The Authors provided raw data for these figures on request from the Editors; however, an investigation by the journal revealed discrepancies between the original and published data. Due to these concerns, the Editors no longer have confidence in the work presented in this Article.

Author V. Puneeth disagrees with the retraction. Authors Shuguang Li, A. M. Saeed, A. Singhal, Fuad A. M. Al-Yarimi, M. Ijaz Khan, and Sayed M. Eldin have not responded to correspondence from the Editors about this retraction.

